# Feasibility analysis of an ultrasound on line diagnostic approach for oral and bone surgery

**DOI:** 10.1038/s41598-022-04857-0

**Published:** 2022-01-18

**Authors:** Maria Alessandra Cutolo, Carlo Cafiero, Luigi Califano, Martino Giaquinto, Andrea Cusano, Antonello Cutolo

**Affiliations:** 1 Regional Center on Information Communication Technology (CeRICT) scrl, 82100 Benevento, Italy; 2 Department of Neurosciences, Reproductive and Odontostomatological Sciences, University of Naples “Federico II”, 80131 Naples, Italy; 3 Optoelectronics Group, Engineering Department, University of Sannio, 82100 Benevento, Italy; 4 Department of Electrical Engineering and Information Technology, University of Naples “Federico II”, 80121 Naples, Italy

**Keywords:** Biomedical engineering, Dental diseases

## Abstract

During implant surgery procedures, surgical precision is an essential prerequisite for the functional and aesthetic success of the prosthetic crown to be placed on the dental implant. A modern implant surgical approach should be standardized as much as possible to guarantee extreme precision in the insertion of the implant into the upper and lower bone jaws. Among the most common surgical errors during implant surgery there is the over-preparation of the surgical alveolus with possible damage to the contiguous anatomical structures. To avoid this problem, in the recent years, there has been an increasing attention to the development of new control techniques. In this paper, we describe an innovative ultrasound approach, which exploits the integration of an electro-acoustic transducer with the surgical drill used for realizing the alveolus in the bone that will host the implant. Specifically, he proposed approach is based on the “time-of-flight” detection technique for measuring the thickness of the residual bone subjected to the drilling. In order to demonstrate the feasibility of the proposed approach, here we report on a detailed numerical analysis aimed at studying the propagation of ultrasonic waves through the drill-bit and through the involved tissues. The obtained results confirm the validity of our approach, and enable for a future first prototype implementation of a hi-tech surgical drill-bit, which in general is suitable not only for dental implant surgery but also for other uses in oral surgery, maxillofacial surgery and for bone surgery.

## Introduction

Implantology is an oral surgery technique designed to replace lost teeth with a fixed prosthesis^1^. It involves the insertion into the jawbones of dental implants typically made of titanium or other biocompatible materials^2–6^. The implant is characterized by the presence of accessory retention elements, such as thread necessary for primary retention^7^. Some months after dental surgery the implant osseointegration will take place: it means that the inserted implant and the surrounding bone will have a tight contact with no continuity solution^8^. The implant is utilized as an “artificial dental root” in order to support a tooth-shaped prosthetic replacement, called “dental crown”. The surgical technique for the insertion of the implant involves the use of a surgical drill typically completed with sequentially burs that, under constant irrigation with pre-cooled saline solution, make a hole in the bone, which represents the surgical site in which the implant will be placed^8^. The control of the drilling depth in the jaw bone is instrumental to avoid to damage the anatomical structures adjacent to the implant site. The drilling depth of the jaws bone is an essential step in implant therapy. In fact, during the drilling process, delicate anatomical structures (such as adjacent teeth, maxillary sinus, nasal coanas, lower alveolar nerve, etc.) might be damaged by the use of the rotating burs. Implant surgery is generally “a freehanded technique” carried out by operators. This execution is extremely operator-dependent and, therefore, not free from intra-surgical errors with particular reference to the drilling depth of the bone, the axis of implant insertion and the correct distance between implant and adjacent anatomical structures. In view of the previous considerations, and in order to avoid implant insertion errors, in recent years, there has been an increasing attention to the development of new control techniques. In particular, digitized procedures have been developed with the following main steps^9–15^:

1. Pre-implant prosthetic design, (using Laboratory Prosthetic Design Software);
2. Construction of the radiological guide (the patient will be submitted to computed tomography wearing this guide).
3. Three-dimensional reconstruction of the dental arch.
4. The implant surgical project processed in 3D is imported into the laboratory prosthetic modeling software for the fabrication of the surgical guide using CAD/CAM technology, in order to control drilling depth, distance between implants and adjacent teeth as well as the orientation of the implant insertion axis.

Even if they are very precise, these procedures are very time and money consuming and, therefore, they are not suitable for routine implant surgery. In fact, digital design techniques are preferred in complex cases when the insertion of multiple implants in arches with little bone tissue available is required. For these reasons, although computer-guided surgery is emerging as a promising approach for ensuring the safety during the procedures, "free handed technique" is still widespread for implantology. In the most frequent cases (e.g. dental practice routines), the techniques currently used to control penetration depth are complicated by blood or other biological fluids invading the operatory field. The control of the drilling depth shows various critical points. The free-hand control is performed by the use of millimeter burs that allow (through the presence of notches placed at a known distance between them) to verify the length of penetration into the bone and confirmed by the use of special depth gauges (essentially small cylinders calibrated with the presence of notches) inserted in the bony hole to measure its depth. During surgery, therefore, in order to avoid damage to surrounding structures, the operator is obliged to periodically stop the drilling phase in order to verify the reached depth of the drilling, using the techniques described above. This control phase is not smooth since it may be affected by errors due to the fact that the surgical environment is generally invaded by blood and saliva. Typically, the desired depth is reached by using the first bur, called "pilot bur", creating the surgical site (called "pilot hole") of the precise length of the implant to be inserted, whose diameter will be subsequently defined using the sequential drills. In order to avoid surgical errors due to the free-hand work, currently, the most implant systems propose a system of mechanical “drill stops” consisting of a series of rings increasing with the diameter of the implant burs that gradually prepare the surgical site^16^. The rings are able to mechanically stop the penetration of the implant burs to the desired depth, in order to avoid surgical complications associated with injury to anatomic structures such as mandibular canal, mental foramen, anterior loop of the mental nerve and submandibular fossa in lower jaw and maxillary sinus, mandibular incisive area and the cortical bone under the nose in the upper jaw^17–20^. More recently an auto-stop drilling device for implant site preparation characterized by an eccentric sensor that automatically stops the drill upon contact with soft tissues was proposed^21^.

On this line of argument, we propose an innovative control system, directly integrated with the surgical drill. Such a system, currently patent pending^22^, exploits the use of an ultrasonic approach for on-line monitoring the drilling depth, thus allowing for a safe and fast routine implant. Specifically, the proposed approach relies on the detection of the time of flight of an ultrasonic wave traveling through the bone subjected to the drilling, thus exploiting the same principle that is widely used in medicine for non-invasive diagnostics. This approach is thought to be effectively implemented by exploiting the integration of an electro-acoustic transducer (employed for generating and detecting ultrasonic waves) on the surgical drill typically used in traditional implantology. In other words, thanks to the use of ultrasound based approach, it is possible to equip the driller with an add-on monitoring system, without changing the typical operation procedure of the surgeon, but increasing the safety. Interestingly, the proposed approach can be implemented in computer aided implant surgery in combination with the surgical guide. The presence of an ultrasonic sensor, able to evaluate in real time the residual bone thickness during bone perforation, integrated in computerized implant surgery systems, allows in fact to add further safety and precision to the implant surgical site preparation.

As a proof of principle demonstration of the proposed approach, in this work we present an exhaustive analysis, based on numerical simulations, aimed at studying the propagation of ultrasonic waves through the drill-bit and through the bone, bringing out the critical issues related to the choice of the acoustic frequency, instrumental for the design step. Specifically, the aim of our analysis is to study the acoustic wave propagation through the typical drill bits used in implant surgery, with particular reference to the pilot drill bit, without changing the common operation conditions characterizing the implant surgery procedure. In other words, our goal is to find a suitable acoustic frequency that allows for implementing the proposed approach without requiring a new design of the drill bits. The drill bits, in fact, are already optimized in terms of geometry and materials used for creating the surgical hole, and the acoustic wave generated for monitoring the drilling must not interfere with the typical operation of the surgical drill. This can be achieved by using low acoustic powers, which analogously to the common imaging technique based on the use of ultrasounds, allows to detect the waves reflected at the interface between the bone and the soft tissues, without causing strong vibrations which would cause the damaging of tissues, and even more of the drill bit.

## Results and discussion

### The basic configuration

According to the previous discussion, one of the central problems for an efficient and safe implant surgery is the exact knowledge of the residual thickness of the bone during the drilling process. To face this issue, as reported in the previous section, we have looked for a simple and user-friendly solution, which consists in an innovative device relying on an ultrasound approach. This solution can increase both the safety and the efficiency of many oral surgery applications. It is essentially based on the use of a piezoelectric crystal mounted in proximity of the drill-bit in order to perform an on line measurement of the thickness of the residual bone in front of the drill. This technique may be instrumental for other bone surgery as well. This idea came out while working on a different research project finalized to the attempt to integrate a novel ultrasound approach for selective deletion of cancer cells^22,23^ inside a new theranostic technological platform: the hospital in the needle^24–29^.

**Figure 1:**
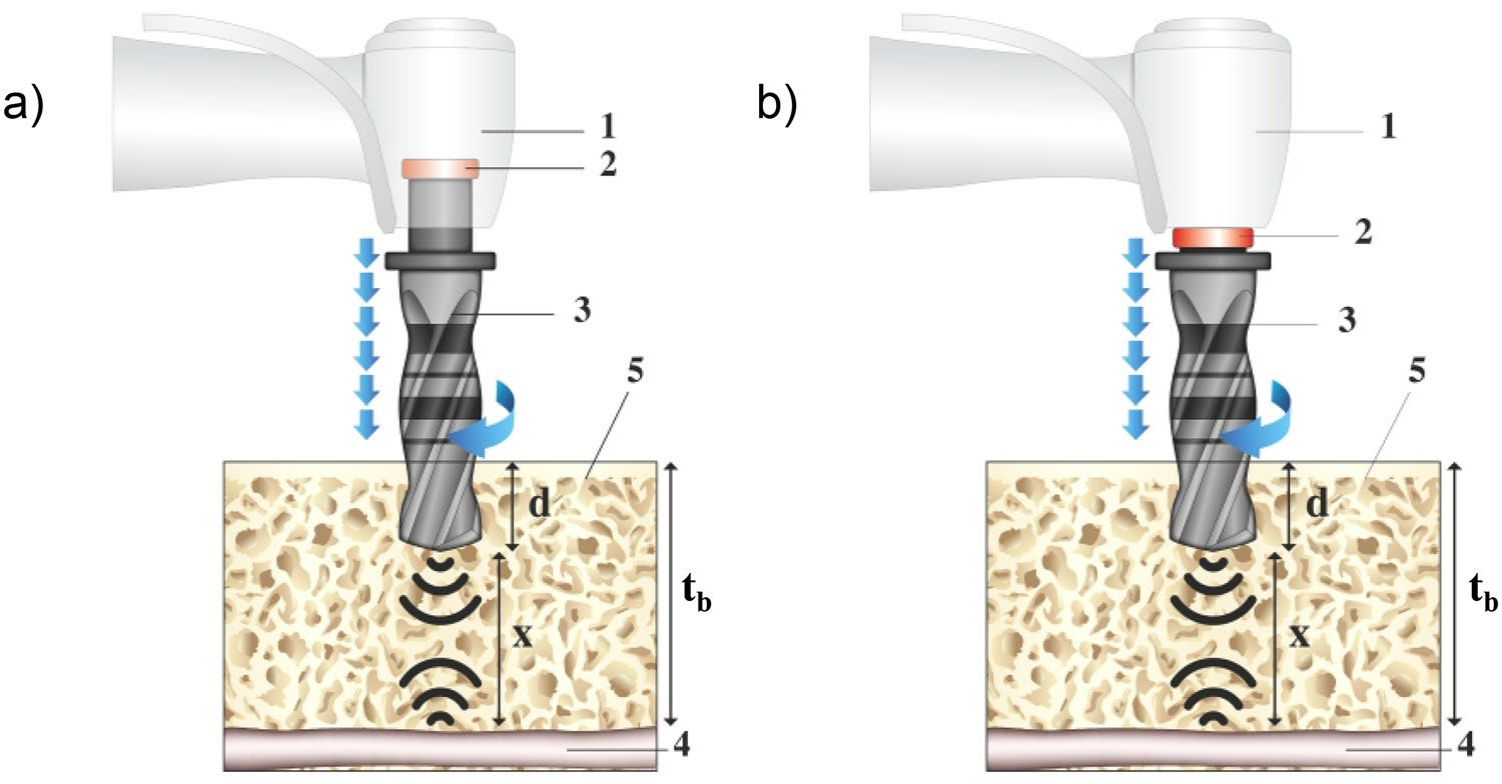
Schematic representation of the proposed system composed of a handful (1), an electro-acoustic transducer (2), a drill-bit of metallic material (3). Specifically, the device is shown in two different configurations: with the electro-acoustic transducer mounted on the static component (**a**) and on the rotating component (**b**). In both the cases, the transducer (that can be a piezoelectric crystal) works as generator and detector of acoustic signals. The regions (4) and (5) in (**a**) and (**b**) represent the soft tissue and the bone, respectively.

The proposed device is schematically represented in Fig. 1. Specifically, the device is thought to be implemented in two different configurations, i.e. the first one (Fig. 1a) with the transducer integrated on the static component of the system, and the second one (Fig. 1b) with the transducer integrated on the rotating component. In both the cases, the transducer (that can be a piezoelectric crystal) works as generator and detector of acoustic signals. In more details, with reference to the schematics of Fig. 1a,b, the device is composed of a handful (1), an electro-acoustic transducer (2), a drill-bit of metallic material (3). Figure 1 also shows the soft tissue (4) and the bone (5) that will host the implant. Moreover, “t_b_” is the total thickness of the bone that is typically known thanks to radiographic examinations. Finally, the length “x” represents the residual thickness of the tissue subject to drilling, while “d” is the penetration depth of the drill-bit.

With a look toward the real implementation of the proposed system, we refer to two possible alternative configurations, in order to take into account the problem linked to the electrical connections between the transducer and the control system of the same (including the power supply), although these issues goes behind the scope of this work.

Independently on the configuration, the surgical drill integrated with an electro-acoustic transducer allows for the direct measurement of the length “x” by exploiting the detection of the “time of flight”, i.e. the time necessary for an ultrasonic wave to propagate along the distance “x”. In more details, the ultrasonic signal generated by the transducer propagates through the metallic drill-bit, to be then irradiated into the bone. When the wave reaches the discontinuity between the two tissues (i.e. the bone and the soft tissue), part of it will be back reflected and will travel in the opposite direction (through the bone and through the drill-bit), thus impacting on the transducer after a time delay strongly depending on the path length.

In order to clarify the real feasibility of our idea it is necessary to study how the vibrations produced on the drill-bit will propagate through the bone, to be then coupled back to the transducer, thus allowing for the time of flight detection. More specifically, it is important to understand how the transducer frequency vibrations would influence the effective capability of the proposed system of detecting the reflected wave, also looking for possible weak points of our approach. It is worth to consider that the problem to find a suitable acoustic frequency is strongly related to the instrument operation, because its value must satisfy two basic counter posed design criteria. First, the acoustic frequency must be high enough to guarantee the appropriate spatial resolution. The spatial resolution, in fact, is determined by the acoustic wavelength, which is inversely proportional to the frequency^30^. On the other hand, the attenuation of an acoustic wave is proportional to the square of its frequency^31–33^. This means that high frequencies would require high acoustic power densities in order to avoid a reduction of the signal-to-noise ratio. The feasibility of our idea rests hence on the possibility of using an acoustic frequency that satisfies all the requirements for the efficient and safe use of the proposed device. On the base of these observations, we specifically focused our attention on the “time of flight” detection through a measurement of the phase delay between the generated and the reflected acoustic waves. This approach allows to detect the time of flight working with acoustic wavelengths higher than the residual bone thickness, thus enabling the use of working frequencies that does not exceed the range of hundreds of kHz^30^. This frequency range would allow to reach the desired resolutions (i.e. measuring thicknesses in the order of a few millimeters with an accuracie around 0.1 mm), also limiting issues related to the attenuation of acoustic frequencies during the propagation. The phase delay can be measured by exploiting standard methods, such as those based on the use of a counter (event-counter methods), those involving the use of a frequency mixer to multiply the signal with another one from a reference oscillator (modulation-based methods), or those that exploit the signal sampling to then retrieve the unknown phase through a post-processing (sampling-based methods)^34^.

Moreover, this range of frequency is easy to handle from different points of view including the signal processing needed to determine the residual thickness. Given these considerations, we need to check if this frequency range can comply with any propagation problem without requiring high power levels. For this purpose, we carried out a numerical analysis based on a reliable model, experimentally validated in a previous work^35^. The results of this analysis are discussed in the next section.

### Numerical analysis

**Figure 2:**
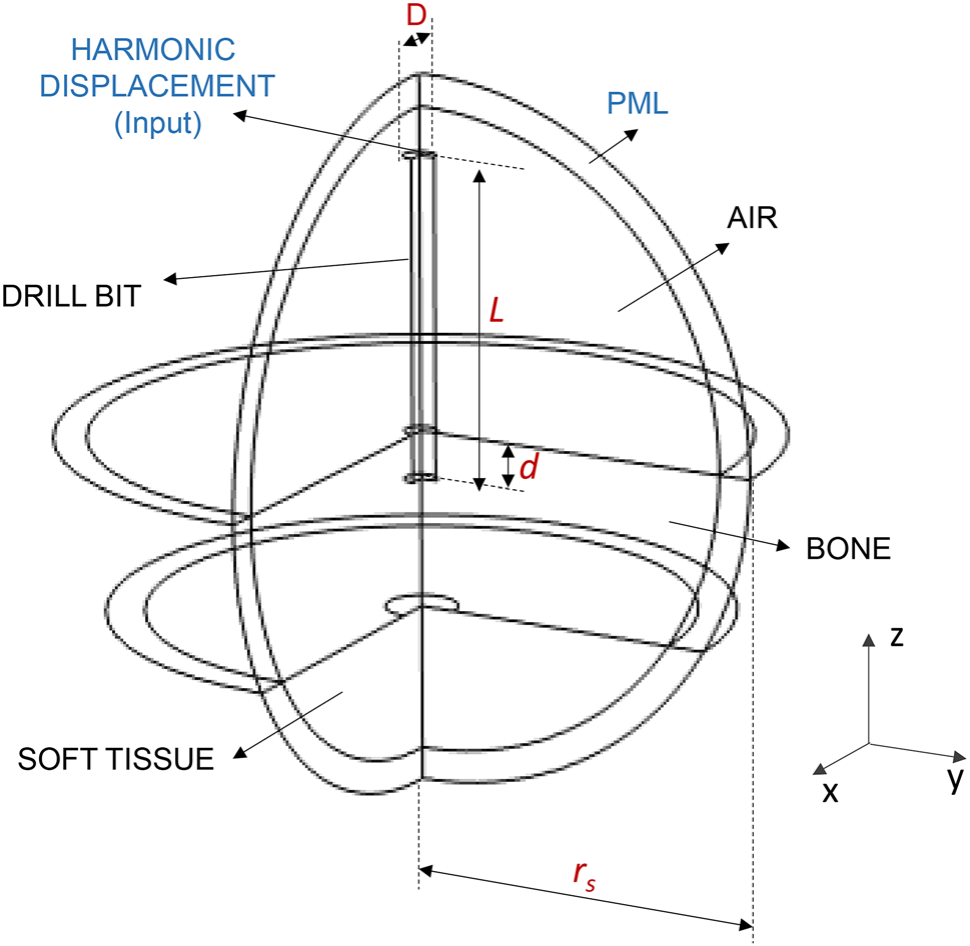
Schematic representation of the simulation domains.

In this section, we report on the main results of a numerical analysis carried out with reference to the basic schematic of the proposed device shown in Fig. 1. In more details, the propagation of ultrasonic waves from the drill bit to the bone/soft tissue interface was simulated by using the commercial software Comsol Multiphysics, based on Finite Element Method (FEM)^36^ where the drill bit and the tissues (soft and bones) were modeled as linear elastic media. The simulation domains are schematically shown in Fig. 2.

The drill bit is modeled as a cylinder of stainless steel (Young modulus E_db_ = 205 GPa, Poisson ratio ν_db_ = 0.28 and density ρ_db_ = 7850 kg/m^3^, according with^37^) characterized by a length *L* = 2 cm and diameter *D* = 2 mm.

The bone is modeled as a homogenous solid domain (of thickness equal to 10 mm), with Young modulus E_b_ = 17 GPa, Poisson ratio ν_b_ = 0.3 and density ρ_b_ = 1908 kg/m^3^. These values averagely describe both the cortical bone^38^ and the trabeculae constituting the structure of the cancellous bone^39,40^. A more detailed discussion about the bone properties is provided in “Discussion about the non-idealities”.

The soft tissue, instead, was treated as a fluid with density of 1 kg/m^3^ and speed of sound of 1540 m/s (neglecting the frequency-dependent attenuation effects)^41^. The whole simulation domain was terminated with a spherical surface with radius *r*_*s*_, kept constant at 4 cm during all the simulations, at which a perfectly matched layer (PML) was applied for avoiding the reflected components at the boundaries, in such a way to mimic an infinitely extended tissue. A harmonic longitudinal displacement, with frequency *f* and amplitude *A*, is applied at the top boundary of the drill bit; this condition constitutes the input stimulus applied to the simulated geometry. On the rest of the drill bit boundaries (placed in contact with air, bone and soft tissue) a proper condition is imposed, by considering that the adopted model involves the coupling between two physics, one pertaining to structural mechanics (for the drill bit and bone domains) and one to acoustics (for the soft tissue domain)^35,36,41–43^. More details about the numerical model are described in methods section, while a critical discussion about the non-idealities is provided in “Discussion about the non-idealities”.

**Figure 3:**
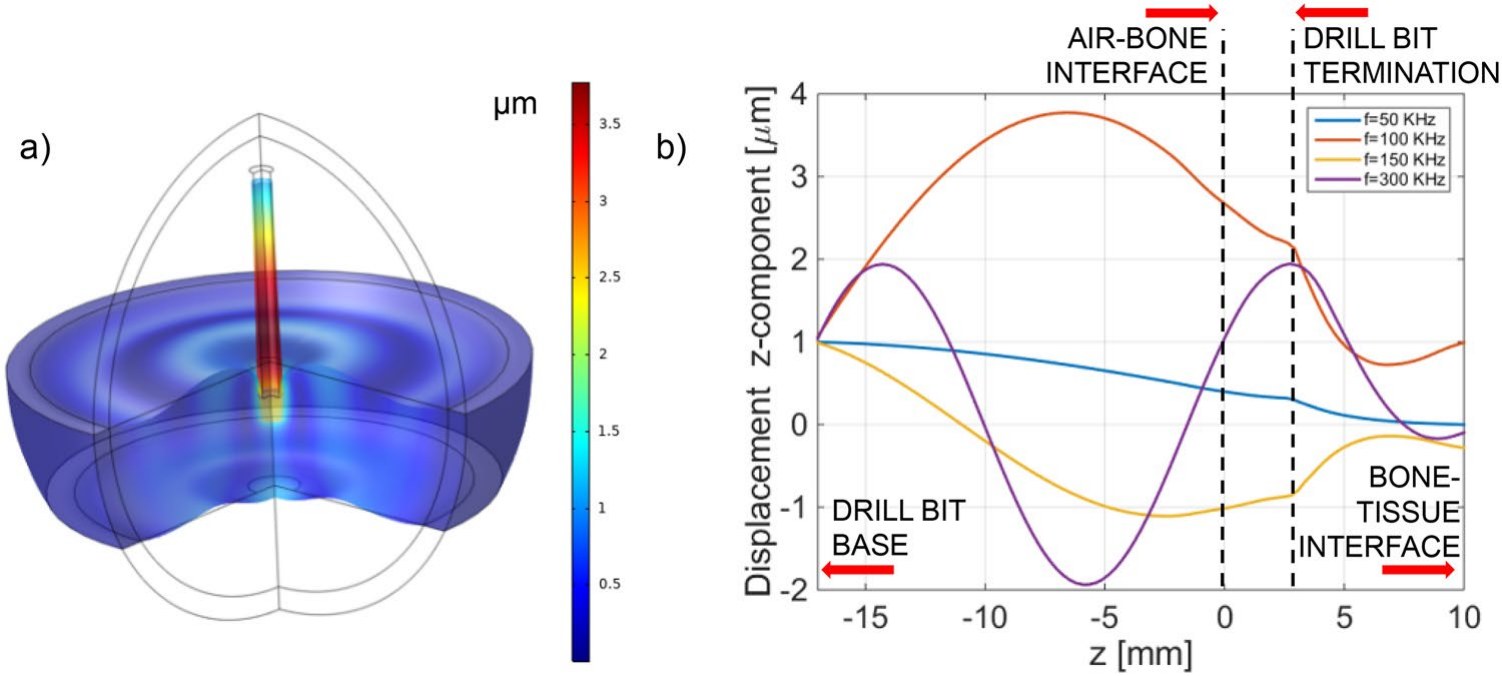
(**a**) Total displacement field evaluated at 100 kHz. (**b**) Longitudinal displacement along the drill bit axis of symmetry evaluated at different frequencies.

As a first analysis, we evaluated the total displacement field in the solid regions (drill bit and bone) by setting, without loss of generality, the input amplitude *A* and the frequency *f* equal to 1 μm and 100 kHz respectively, while the considered penetration depth of the drill bit inside the bone is *d* = 3 mm. The results represented as a 3D map in Fig. 3a show a displacement that assumes its maximum inside the drill bit and rapidly decreases inside the bone region.

With the aim of selecting the working frequency, we evaluated the longitudinal component of the displacement (z-component) along the drill-bit symmetry axis, up to the interface between bone and soft tissue, for different frequencies of the harmonic displacement applied at the drill bit base. The results, shown in Fig. 3b, are discussed by considering the ratio between the acoustic wavelength and the propagation lengths in the single regions.

At the higher analyzed frequency (300 kHz) the displacement assumes a sinusoidal-like oscillating behavior. In this case, the acoustic wavelength (evaluated by considering the speed of the longitudinal waves) is comparable with the propagation lengths into the two solid domains (about 1.7 cm in the drill bit and 1 cm in the bone). By decreasing the working frequency, the acoustic wavelengths in the drill bit and in the bone consequently increase. In other words, the spatial period of oscillation increases and tends to become larger than the propagation lengths. Consequently, at the frequency equal to 50 kHz, for what the wavelengths are about 5-folds higher than the drill bit lengths and bone thickness, the displacement appears approximately uniformly distributed into the domains^30,44^.

The selection of the working frequency should to take into consideration the modality that will be adopted for the evaluation of the time of flight. If the time of flight is evaluated by measuring the phase shift between the input wave and the reflected wave, it is worth to avoid oscillations inside the bone and the drill bit. For this reason, the choice of a low frequency (for instance 50 kHz) would be preferred, while higher frequencies such as 300 kHz should be avoided. However, the amplitude of the vibration induced at the bone/tissue interface is also an important parameter to take into account, since the coupled back wave, have to be detected by the sensor placed at the drill bit base, and should be enough high to be transduced into an electrical signal. For this purpose, in order to estimate the amplitude of the displacement induced by the back-propagating wave on the drill bit base, in first approximation, we carried out a second simulation, in which we turned off the input applied at the drill bit base. In this second simulation, we considered as input signal the vibration at the bone/soft tissue interface evaluated with the previous simulation step, then evaluating the average longitudinal displacement induced at the drill bit base. More specifically, we performed the same simulations for different penetration depths, from 1 to 6 mm.

**Figure 4:**
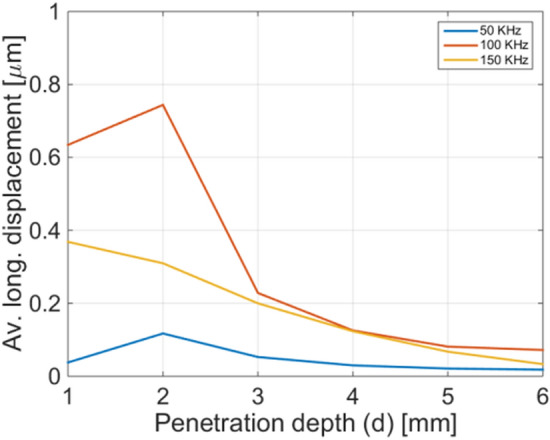
Average longitudinal displacement induced at the drill bit base by the reflected wave, considering an input displacement of 1um.

The results plotted in Fig. 4 show an almost decreasing trend of the average longitudinal displacement as a function of the penetration depth. This phenomenon could be explained by considering that the higher is the portion of the drill bit introduced in the bone region, the higher is the damping induced to the vibrations. Overall, these results demonstrate that, in correspondence of an input vibration with amplitude of 1 μm applied at the drill bit base, the coupled back wave induce a vibration, at the same position, with amplitudes in the order of hundreds nanometers. More specifically, the amplitude is lower at 50 kHz, due to a lower amplitude of the incident wave at the bone/tissue interface (blue curve of Fig. 2b), while assumes maximum values at 100 kHz, coherently with the higher displacement induced at the bone/soft tissue interface (orange curve of Fig. 2b). On the base of these considerations, frequencies around 100 kHz result as good trade-off between the amplitude of the vibration read by the sensor placed at the drill bit base, and the ease of flight time measurement.

### Discussion about the non-idealities

In our analysis, aimed at demonstrating the feasibility of the proposed approach, we have considered different assumptions, which if from one hand allow to simplify the problem, thus focusing on the essential aspects of the feasibility analysis, on the other hand they may limit the application of this model for an effective design of the device. For this reason, it is worth to shade light of some non-idealities, which will be taken into account in a future work aimed at designing and experimentally testing a first prototype of the proposed device.

In this regard, the modeling of the bone deserves some important considerations. The mandibular bone is constituted by both cortical and cancellous (or trabecular) components and its characteristics are strongly dependent on the patient. In fact, according with Mish, the bone density can be classified as follows^45^: D1 is dense cortical bone, D2 is porous cortical and coarse trabecular bone, D3 is porous cortical bone (thin) and fine trabecular bone, and D4 is fine trabecular bone. Bone has a ten-fold difference in strength and flexibility between D1 and D4 bone qualities^46^. This means that, generally, the bone has a non-homogenous structure, with mechanical properties that depends on the bone quality. More specifically, the bone Young Modulus can change from ~ 0.3 GPa (for cancellous bone) to ~ 20GPa (for cortical bone), while its density can vary in a range between ~ 1000 and ~ 2100 kg⁄m^3^^39,40^. According to the Biot’s theory, the fluid-saturated cancellous bone supports the excitation of two waves, namely the ‘fast’ (or first order) and the ‘slow’ (or second order) wave. The fast wave has a velocity that spans over a range between ~ 2500 and ~ 3500 m/s, while the slow wave can change between ~ 1000 and ~ 1400 m/s^47,48^. For this reason, the amplitude and the phase delay of the wave reflected at the interface bone/soft tissue and detected at the drill bit upper termination may be affected by the bone characteristics. While the amplitude variation does not affect the bone thickness estimation, the variation on the phase delay may affect the measurement precision. However, it is important to remark that the characteristics of the bone subjected to the drilling are typically known from preliminary analysis, conventionally used in implantology (such as those based on TAC or RX). In addition, since the amplitude of the detected signal changes according with the characteristics of the bone, this information may be in principle used for a self-calibration of the device. Moreover, it is also worth to consider that the solid mineralized trabecular network could give rise to scattering phenomena, thus introducing strong attenuation to the acoustic waves propagating through the bone. In this regard, it is important to underline that the scattering is strongly dependent on the acoustic wavelength and thus on the operating frequency^32,49^. The mechanical properties used in our model, averagely describe both the cortical bone and the trabeculae constituting the structure of the cancellous bone^39,40^, giving rise to a speed of sound c_b_ = 2985 m/s, which, without loss of generality, well describe the waves propagating in cortical bone and the fast wave propagating in the wet cancellous bone^47,48^. This velocity value corresponds to a wavelength of about 30 mm at a frequency of 100 kHz, therefore, in the range of frequencies considered in this analysis, the elements constituting the cancellous bone have a dimension much smaller that the acoustic wavelength. Based on this consideration, we considered negligible the scattering effects^32,49^, modeling the bone as a homogenous medium.

Concerning the soft tissue, in our model we considered for it a ‘very extensive’ region, which is essentially the ‘simplest’ condition, since only one reflective interface is present, namely the discontinuity between the bone and the soft tissue. This condition allowed us to evaluate the amplitude of the reflected wave coupled back to the transducer placed to the upper end of the drill-bit. In a more realistic scenario, the propagating wave will experience multiple reflections at the different discontinuities between bone and soft tissues, giving rise to multiple reflected components in the detected signal. However, it is reasonable to think that the back propagating wave generated at the first discontinuity has a lower phase delay (and also a different amplitude) with respect to the waves reflected at more distant discontinuities. Therefore, the detection/elaboration system (whose definition is out of the scope of this work, and will be addressed in a future study) could be in principle able to discriminate among the different received contributions, also providing information about the stratification of the tissue subjected to the drilling.

Finally, it is worth to take into account that the geometry of a realistic drill bit is more complicated than a ‘simple’ cylinder, due to the presence of the thread, which essentially makes its surface uneven and not perfectly adherent to the walls of the hole inside the bone. This irregularity, may give rise to scattering effects at the lateral surface of the drill bit, which are expected to be more significant when their feature size become comparable with that of the acoustic wavelength in the drill bit. Scattering effects essentially introduce losses along the propagation of the vibration wave, with a consequent reduction of the signal amplitude read by the detector, but without affecting the phase delay.

## Conclusions

In this paper, we have presented the feasibility analysis of an innovative and very simple approach for on line and real time monitoring the drilling process during implant surgery. The proposed approach is based on the detection of the time of flight of an ultrasonic wave generated at the top of the drill bit, allowing for the measurement of the residual thickness in front of the driller. Our analysis, based on numerical simulations, which describe the propagation of ultrasonic waves through the drill bit and through the involved tissues, suggests that frequencies around 100 kHz are suitable to this goal. The results in fact demonstrate that at this frequency range, the acoustic waves reflected at the interface between the bone and a soft tissue are coupled back to the drill bit and reach the transducer, thus allowing for their detection. Specifically, at 100 kHz the amplitude of the vibration caused by the reflected wave is maximized, while the displacement distribution in the residual bone thickness and in the drill bit is suitable for detecting the time of flight by exploiting a phase delay measurement^50^. Moreover, it is important to point out that at 100 kHz the attenuation of ultrasonic waves in the tissues are limited^31^. Overall, the discussed numerical analysis demonstrates the feasibility of the proposed approach, and lays the groundwork for the effective implementation of a surgical drill equipped with an electro-acoustic transducer. Future studies will be devoted to design and implementing a first prototype, in such a way to experimentally validate the approach. Specifically, the device can be essentially implemented in two different configurations, which also take into account the electrical connection of the transducer. When the transducer is mounted on the static part, the propagation of the ultrasonic signal is more complex (due to the presence of different discontinuities and the longer propagation path) but the electrical connection is simpler. This solution allows for the monitoring of the residual thickness to be drilled in real time and continuously during the intervention. On the other hand, if the transducer is mounted on the rotating part, the ultrasonic wave propagation is more efficient, but the connection problem can be more complex, opening the way to two possible scenarios. The first involves the use of sliding contacts to ensure to the operator the availability of the measurement with continuity. Considering the high rotation speed of these drills, a direct consequence could be the aging to which the contacts are subjected. The other possibility is the use of connectors to be linked only during the measurement phase. In more details, the transducer can be kept electrically disconnected from the control system during the drilling phase. When it is necessary to check the depth reached, the operator can stop the rotation, and electrically connect the transducer through a connector. This operation allows checking the depth reached without extracting the drill-bit from the surgical site drilled. After the control, the operator can disconnect the transducer for starting again the drilling phase. Despite the need to temporarily interrupt the drilling phase, the whole process is simpler than any other possibility currently offered by the market.

This proposed device appears to be very promising in the context of high-precision implant surgery after careful diagnostic examination. In fact, this instrument has the potential to carry out, during implant surgery, the prevention of lesions of the anatomical structures surrounding the implant site (nerves, maxillary sinus, nasal coana, contiguous teeth). The extreme precision of implant insertion, in fact, is an essential prerequisite for the functional and aesthetic customization of the prosthetic crown that will be cemented or screwed onto the implant itself.

## Methods

### Numerical model

The small vibrations applied at the drill bit base causes small stress perturbations, therefore, assuming that the drill bit behaves as an elastic medium, the displacement vector ***U*** in the solid domains can be evaluated through the following elasto-dynamic equilibrium (Navier’s) Eq. (1), as reported in^36,49^.

1 $$\mu {\nabla }^{2}{\varvec{U}}+\left(\lambda +\mu \right)\nabla \left(\nabla \cdot {\varvec{U}}\right)+{\varvec{F}}={\rho }^{^{\prime}}\frac{{\partial }^{2}{\varvec{U}}}{{\partial t}^{2}}$$

being **F** the dynamic load applied at the drill bit base, $\rho$ the density of the involved materials, $\lambda$ and $\mu$ the Lamè constants expressed as a function of the Young’s modulus E and Poisson’s ratio ν as follows:

2 $$\lambda =\frac{\mathrm{\nu E}}{(1+\upnu )(1-2\upnu )};\mu =\frac{\mathrm{E}}{2(1+\upnu )}$$

Knowing the displacement, it is possible to also evaluate the Cartesian components ${\varepsilon }_{ij}$ of strain tensor **ε**, which in the assumption of small perturbation is given by the Eq. (3) according with^35^

3 $${\varepsilon }_{ij}=\frac{1}{2}\left(\frac{\partial {U}_{i}}{\partial j}+\frac{\partial {U}_{j}}{\partial i}\right)$$

which in turns provides the stress vector **σ** by means of the generalized Hooke law (4):

4 $${\varvec{\sigma}}={\varvec{D}}\cdot {\varvec{\varepsilon}}$$

being **D** the elastic constant matrix^43^

The structural vibrations of the drill bit are transferred to the bone, and thus to the soft tissue*.* In the assumption that nonlinear effects in tissues can be neglected and that the amplitude of shear waves is much smaller than that of pressure waves, the following Helmholtz Eq. (5) has been considered for modeling the acoustic waves propagation inside the soft tissue^41^:

5 $${\nabla }^{2}p+\left(\frac{2\pi f}{c}\right)p=0$$

where *p* and f are the acoustic pressure and frequency *f*, respectively.

Consequently, in order to couple the two physics, it is necessary to impose the continuity of the normal displacement at the solid/fluid interfaces, the static equilibrium between the pressure and the stress normal to the solid boundaries, while the tangential stresses at the fluids boundaries must be zero^42^.

### Ethics approval and consent to participate

This manuscript does not report studies involving human participants, human data or human tissue must.

### Consent for publication

This manuscript does not contains any individual person’s data.

## Data Availability

All data generated or analyzed during this study are included in this published article.
